# Identifying Structural Domains and Conserved Regions in the Long Non-Coding RNA lncTCF7

**DOI:** 10.3390/ijms20194770

**Published:** 2019-09-26

**Authors:** Michael C. Owens, Sean C. Clark, Allison Yankey, Srinivas Somarowthu

**Affiliations:** Department of Biochemistry and Molecular Biology, Drexel University College of Medicine, Philadelphia, PA 19101, USA; michael.carl.owens@gmail.com (M.C.O.); clarsc9@gmail.com (S.C.C.); allison2yankey@gmail.com (A.Y.)

**Keywords:** long non-coding RNAs, lncTCF7, WSPAR, lncRNA, RNA structure

## Abstract

Long non-coding RNA (lncRNA) biology is a rapidly growing area of study. Thousands of lncRNAs are implicated as key players in cellular pathways and cancer biology. However, the structure–function relationships of these novel biomolecules are not well understood. Recent structural studies suggest that lncRNAs contain modular structural domains, which play a crucial role in their function. Here, we hypothesized that such structural domains exist in lncTCF7, a conserved lncRNA implicated in the development and progression of several cancers. To understand the structure–function relationship of lncTCF7, we characterized its secondary structure using chemical probing methods. Our model revealed structural domains and conserved regions in lncTCF7. One of the modular domains identified here coincides with a known protein-interacting domain. The model reported herein is, to our knowledge, the first structural model of lncTCF7 and thus will serve to direct future studies that will provide fundamental insights into the function of this lncRNA.

## 1. Introduction

Long non-coding RNAs (lncRNAs) are RNA molecules of at least 200 nucleotides in length that do not code for proteins [1]. Despite their lack of coding potential, lncRNAs play critical roles in both cell biology and disease [2,3,4,5,6]. As of 2019, there are 56,946 lncRNA genes deposited in the LNCipedia database, many of which are dysregulated in several diseases, including cancer and viral infections [7,8,9]. Emerging research shows that lncRNAs function as scaffolds for proteins and as “decoy” targets for miRNAs [1,10]. In contrast to our expanding knowledge regarding the function of lncRNAs, the molecular details regarding their mechanisms of action are largely unknown [11,12].

RNA secondary structure determination is an ideal starting point for in-depth mechanistic studies [11,12,13]. An RNA secondary structure map allows for identification of structural domains and motifs that accelerates functional studies [14,15]. However, in contrast to the ever-increasing number of discovered lncRNAs, only a handful of secondary structures have been published, including SRA (steroid receptor RNA activator) [16], HOTAIR (HOX transcript antisense RNA) [17], Xist (X-inactive specific transcript) [18], RepA [19], and Meg3 [20,21]. For a more detailed list of lncRNA secondary structures, see Qian et al. [12].

Given this dearth of structural information on lncRNAs, and the increasing evidence of their biological importance, we have determined the secondary structure of the cancer-relevant lncTCF7 (also known as WSPAR; WNT signaling pathway activating non-coding RNA). LncTCF7 has been implicated in the development and progression of multiple cancers, including liver cancer, colorectal cancer, non-small cell lung cancer, and glioma [22,23,24,25,26,27,28,29,30,31,32,33]. LncTCF7 is transcribed from the locus (5q31.1) upstream of the gene TCF7 (transcription factor 7). Independent studies have shown that lncTCF7 promotes cancer metastasis and tumor growth via activation of the WNT signaling pathway [30,33]. The current model suggests that lncTCF7 recruits the SWI/SNF (mating-type switching/sucrose non-fermentable) complex to the promotor of the TCF7 gene, thus increasing transcription of the TCF7 and subsequently increasing signaling through the WNT pathway [33]. However, the molecular details of how lncTCF7 exerts its function remain poorly understood.

As a first step towards a detailed understanding of lncTCF7 function, we mapped its secondary structure using complementary probing techniques. First, we purified lncTCF7 to homogeneity, using a native purification method [34]. We then probed its structure using a SHAPE (selective 2′-hydroxyl acylation analyzed by primer extension) reagent to obtain a secondary structural model. We next validated our model using shotgun secondary structure (3S) analysis, and an orthogonal probing reagent, DMS (dimethyl sulfate). This combined analysis highlighted two potential regions of interest in lncTCF7, which show high confidence and low Shannon entropy. One of these regions (bases 468 to 683) has been previously shown to recruit the core components of SWI/SNF, suggesting a possible structure–function relationship.

## 2. Results

### 2.1. Purification and Folding of lncTCF7

**Figure 1:**
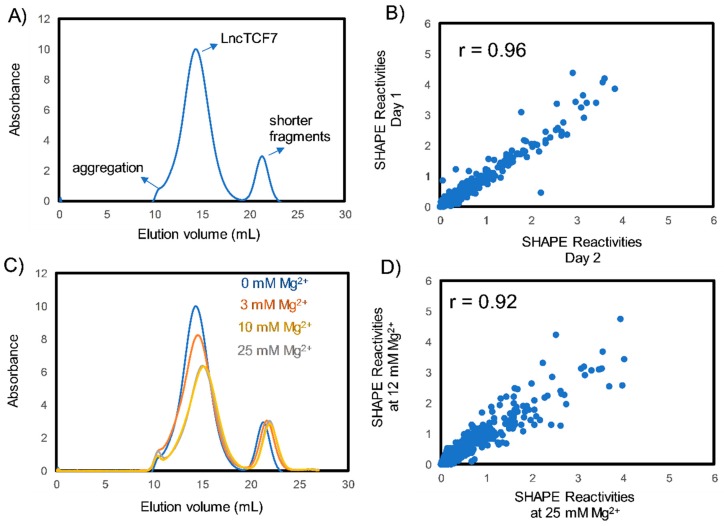
Purification and folding of the lncRNA lncTCF7. (**A**) Size exclusion chromatography (SEC) profile of lncTCF7 purified using native purification. (**B**) Representative scatter plot comparing SHAPE (selective 2′-hydroxyl acylation analyzed by primer extension) reactivities from two independent SHAPE-MaP experiments. Our purification method is reproducible, as indicated by a high correlation coefficient (*r* = 0.96). Experiments were performed in triplicate; correlations were similar between all three replicates. (**C**) SEC profiles of lncTCF7 purified and folded at varying [Mg^2+^]. LncTCF7 can be purified to homogeneity over a broad range of [Mg^2+^]. (**D**) Scatter plot comparing SHAPE reactivities of lncTCF7 folded at 12 mM and 25 mM Mg^2+^ (reactivities represent average of three independent trials). The high correlation (>0.9) between SHAPE reactivities of RNA folded at 12 mM and 25 mM Mg^2+^ indicate that there are no significant structural changes beyond 12 mM [Mg^2+^].

Purification of lncRNAs is a challenging task because of their large size; traditional RNA purification methods involving heat denaturation and refolding often result in misfolding and aggregation when applied on to lncRNAs [17,34]. Therefore, to purify lncTCF7, we have employed a native purification protocol developed by the Pyle laboratory [34]. This protocol preserves the secondary structure formed during transcription and thereby allowed the purification of lncTCF7 to homogeneity (Figure 1A). To test the reproducibility of our purification protocol, we performed SHAPE-MaP (see below) on three independent RNA preparations. The normalized SHAPE reactivities correlated strongly (*r* = 0.96, Figure 1B), suggesting that our purification protocol is highly reproducible and thus suitable for structural studies.

RNA molecules require cations to fold into their native structures [35,36,37]. Divalent cations, such as Mg^2+^, can stabilize RNA structure. However, higher amounts of Mg^2+^ may lead to non-specific aggregation [17]. Therefore, it is essential to identify the optimal Mg^2+^ ion concentration for RNA folding. Here, to identify the optimal [Mg^2+^] required for lncTCF7 folding, we conducted size exclusion chromatography (SEC) at increasing [Mg^2+^] (Figure 1C). The chromatograms obtained by SEC suggest that lncTCF7 can be purified to homogeneity over a broad range of [Mg^2+^]. Increasing [Mg2+] causes a decrease in absorbance (due to the hypochromicity of double stranded RNA) and a rightward shift in the RNA elution volume, both indicating RNA folding and compaction [34]. However, increasing [Mg^2+^] to 50 mM or higher resulted in non-specific aggregation. Nonetheless, the RNA elution peaks perfectly overlap at both 10 mM and 25 mM Mg^2+^, suggesting that RNA folding is not significantly affected by [Mg^2+^] above 10 mM. To test this, we performed SHAPE on RNA folded with 12 mM and 25 mM Mg^2+^ in triplicate (Figure 1D) and observed a high correlation (*r* = 0.92) between SHAPE reactivities at 12 mM Mg^2+^ and 25 mM Mg^2+^, suggesting that there are no significant changes in the secondary structure beyond 12 mM Mg^2+^. Based on this analysis, we used 12 mM Mg^2+^ for probing the structure of lncTCF7, which is the same [Mg^2+^] present in our transcription buffer, and thus keeps the [Mg^2+^] consistent throughout transcription, purification, and folding.

### 2.2. Determining the Secondary Structure of lncTCF7

After establishing the purification protocol and folding conditions for lncTCF7, we next characterized its secondary structure using a SHAPE reagent. SHAPE reagents readily react with the backbone of flexible nucleotides independent of the nucleotide identity and are widely used for RNA secondary structure determination [38]. Recently, the Weeks laboratory developed SHAPE-MaP, a method that combines SHAPE with mutational profiling and deep sequencing for high-throughput determination of RNA secondary structure [39]. Here, using SHAPE-MaP, we measured the SHAPE reactivity of lncTCF7 at single-nucleotide resolution. The normalized SHAPE reactivities were then used as constraints for secondary structure prediction in RNAstructure, which predicted 19 potential secondary structure models for lncTCF7.

#### 2.2.1. Shotgun Secondary Structure Analysis

Next, to identify the correct secondary structure among the 19 possible models, we used the shotgun secondary structure determination method [16,40,41]. In the shotgun approach, the RNA is truncated into smaller fragments, and each fragment is probed alongside the full-length RNA. If the SHAPE reactivities of a given fragment show high correlation with the corresponding region of the full-length, this suggests the presence of an independent subdomain [40]. Identifying such independent subdomains would then allow us to eliminate alternative secondary structure models that do not include these subdomains.

**Figure 2:**
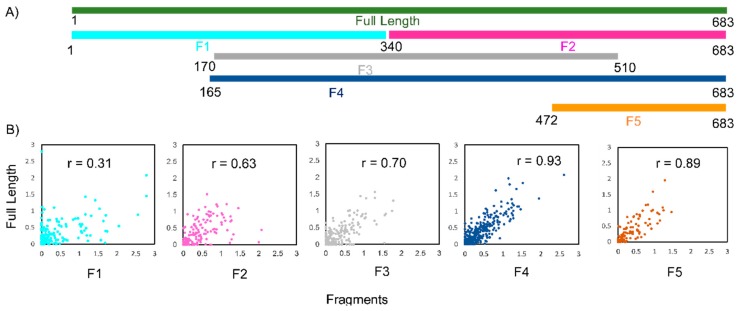
3S (shotgun secondary structure) analysis of lncTCF7 fragments. (**A**) Schematic representing the position of lncTCF7 fragments corresponding to the full-length sequence. (**B**) Scatter plots comparing SHAPE reactivities of each fragment with the corresponding region in full-length lncTCF7. Pearson correlation coefficient (*r*) values are indicated.

**Figure 3:**
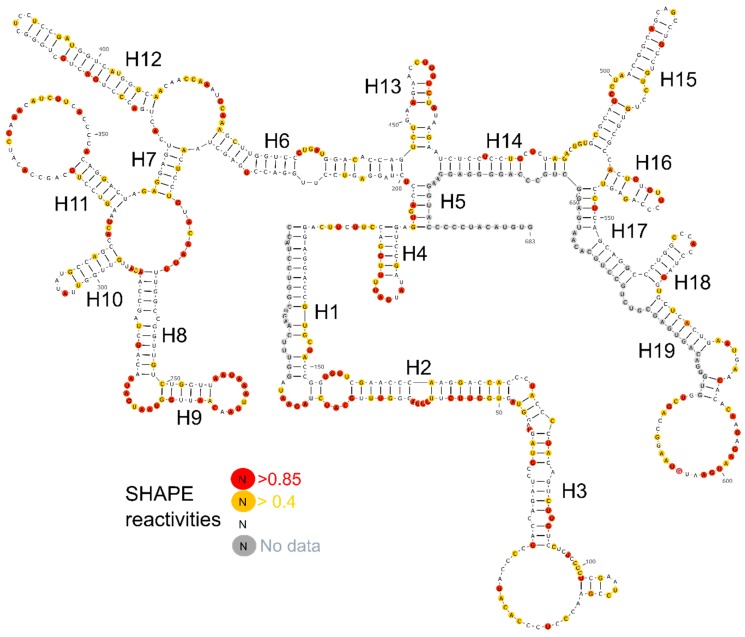
Secondary structure of lncTCF7. SHAPE reactivities are highlighted as depicted in the legend. Nucleotides with high SHAPE reactivity are highlighted in red, nucleotides with medium SHAPE reactivity are highlighted in yellow, and nucleotides with ‘no data’ are highlighted in grey.

We designed five fragments of lncTCF7 spanning various regions: F1 (1–340), F2 (341–683), F3 (170–510), F4 (165–683), and F5 (472–683) (Figure 2A). As with the full-length lncTCF7, we purified all the fragments using SEC. The fragments and full-length were probed in parallel using SHAPE followed by capillary electrophoresis. We compared the normalized SHAPE reactivities of the fragments with the corresponding regions of the full-length using Pearson’s correlation coefficient. Among the five fragments, three fragments showed lower correlation values: F1 (*r* = 0.31), F2 (*r* = 0.63) and F3 (*r* = 0.70) (Figure 2B). This is not surprising, because most of the models predicted by RNAstructure contained long-range base pairs, which are not preserved in fragments F1–3, and thus resulted in lower correlation values. Fragments F4 and F5, however, showed higher correlation values: *r* = 0.93 and 0.89, respectively (Figure 2B). This indicates that the base pairs in these regions are contained almost entirely within the fragment and that they form independent subdomains. Having this information in hand, we examined the models predicted by RNAstructure and identified the secondary structure that is supported by our fragment analysis data. Our model suggests that lncTCF7 is structured, with 56% of the nucleotides base-paired (Figure 3). The model consists of 19 helices, 28 internal loops (13 of which are asymmetric bulges), and 11 terminal loops. Also, the structure contains five higher-order junctions: Two 3-way junctions, and three 4-way junctions (Figure 3).

#### 2.2.2. DMS Probing

To validate the secondary structure model obtained from SHAPE data, we performed DMS-MaP. Unlike SHAPE reagents, DMS methylates single-stranded adenosines and cytidines and thereby serves as an orthogonal approach for probing secondary structure [42,43]. We performed DMS-MaP and collected data for A/Cs in lncTCF7 (Supplementary Figure S1). Overall, 92 nucleotides showed a low DMS reactivity (<0.4), 120 nucleotides showed a reactivity between 0.4 and 0.85, and 123 nucleotides showed high DMS reactivity (>0.85). We found that 81.3% of highly reactive nucleotides are in the loop regions or at the helix termini, indicating that there is a good agreement between our DMS-MaP data and our structural model.

#### 2.2.3. Confidence Estimation

**Figure 4:**
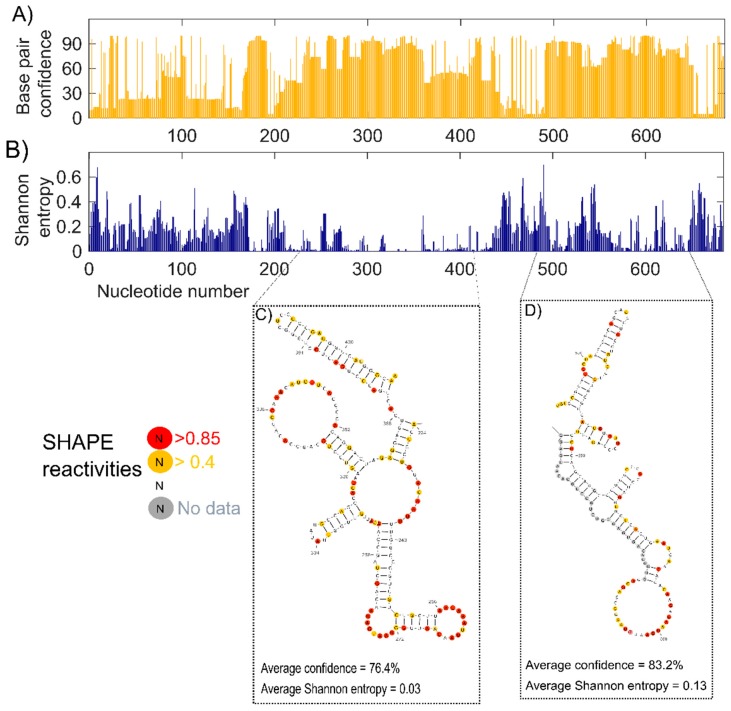
Confidence estimation and Shannon entropy of lncTCF7. (**A**) Confidence estimates of each nucleotide in our structural model of lncTCF7 calculated using jackknife resampling. (**B**) Shannon entropy values of each nucleotide in lncTCF7. Examples of two regions with high confidence and low Shannon entropy are shown in (**C**,**D**). SHAPE reactivities are highlighted as depicted in the legend (see Figure 3 caption for details).

Having identified the secondary structure model that best fits our SHAPE-MaP data, DMS-MaP data, and fragment analysis results, we estimated the confidence of each base pair in our model using jackknife resampling [44]. More than half of the nucleotides showed confidence higher than 70%, and 34.8% of the nucleotides showed confidence below 50%, indicating the presence of both highly structured and dynamic regions (Figure 4A).

### 2.3. Identifying the Well-Defined Structures in lncTCF7

To identify well-defined structural domains in lncTCF7, we calculated Shannon entropy for each nucleotide using RNAstructure (Figure 4B) [45]. Shannon entropy is a measure of conformational entropy. Regions with low Shannon entropy are likely to be highly structured and form stable conformations [45,46]. Such regions often overlap with known functionally important domains [47]. We found several helices in our lncTCF7 secondary structure model with low Shannon entropy (<0.2). A few of these helices also showed high confidence in the jackknife resampling analysis. For example, the region from 489–650 has average confidence of 83.2% and an average Shannon entropy of 0.13. Interestingly, this domain overlaps with the region from 489–683 that is involved in the interaction with the SWI/SNF complex [33]. In addition, we found another region from 224–409, which has average confidence of 76.4% and an average Shannon entropy of 0.03, indicating that this well-defined region is potentially crucial for function (Figure 4C,D).

### 2.4. Identifying the Conserved Regions in lncTCF7

After identifying the structural domains in lncTCF7, we asked whether any of these regions are conserved across mammals. First, using the genomic alignments, we retrieved the corresponding lncTCF7 sequences from all available mammalian species in the UCSC genome browser [48]. Overall, we observed that the full-length lncTCF7 is conserved at the primary structure level with an average percent sequence identity of 61.9% across these 58 mammalian species.

**Figure 5:**
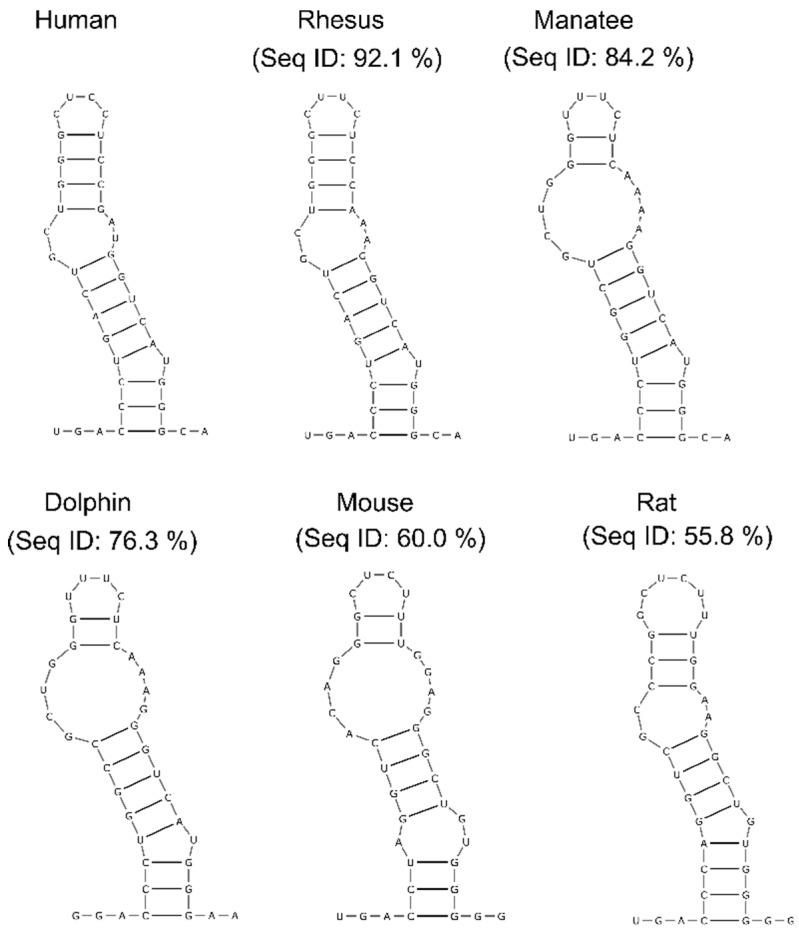
Consensus structures of helix 12 predicted by TurboFold. Percent sequence identities of H12 between human and respective species are indicated.

Next, to identify structurally conserved regions, we used the R-scape (RNA Structural Covariation Above Phylogenetic Expectation) software, which predicts covarying base pairs with statistical significance [49]. R-scape analysis using the highly-sensitive parameter ‘RAFSp’ (average product-corrected RNAalifold with stacking) supported conservation in four helices of lncTCF7: H2, H3, H7, and H12. To test that these predictions were not false positives, we validated the results from R-scape using TurboFold, which predicts common structures among homolog sequences using a combination of thermodynamic folding models and comparative sequence analysis [50]. For the TurboFold analysis, we focused on H12, which has the highest number of significant covarying base pairs: Six out of fifteen base pairs were reported as significant by R-scape. Even without using SHAPE data constraints, TurboFold predicted a conserved structure in H12 among various species, including humans, rhesus macaques, manatees, dolphins, mice, and rats (Figure 5). These combined results suggest that lncTCF7 contains regions that are conserved at both the sequence and structural level.

## 3. Discussion

In the past decades, there has been an explosion in the discovery of lncRNAs, far outpacing the mechanistic studies of these novel biomolecules [9]. Emerging studies show that lncRNAs contain modular domains and motifs that often play critical functional roles [11,51]. For example, domain 1 of HOTAIR can independently bind the PRC2 complex to regulate gene expression [17], though other studies have suggested that this interaction is not necessary for HOTAIR’s regulatory activity [52]. Structural characterization of the lncRNA Braveheart revealed a G-rich motif which recruits cellular nucleic acid-binding protein (CNBP) and thereby regulates cardiomyocyte differentiation [14]. Prompted by these seminal findings, we chose to ask whether the cancer-relevant human lncTCF7 had such domains by characterizing the secondary structure of this lncRNA. Our chemical probing experiments revealed structural domains and conserved regions in lncTCF7, which are potentially crucial for function.

LncTCF7 recruits SWI/SNF to the promoter region of the transcription factor 7 gene and regulates its transcription [33]. Using deletion analysis, previous studies of lncTCF7 identified a region at the 3′-end that is sufficient to pull-down the core components of the SWI/SNF complex [33]. In our structural model, much of this region is structured with low Shannon entropy and high confidence (Figure 4D). Further, we noticed that this region is an independent structural module, meaning that it can maintain its structure even when expressed and folded in isolation (Figure 2). Though our study was not designed to gather information on lncTCF7’s tertiary structure, the structural information we report is nonetheless valuable as it can serve as a guide to design constructs for 3D structural studies and gain additional insights into interactions between lncTCF7 and the SWI/SNF complex.

Structural conservation of RNA helices is a strong indication that these regions are essential for an RNA’s function. However, identifying structurally conserved regions in lncRNAs has been a challenging task [53]. As discussed in our previous study, several factors affect the covariation analysis of lncRNAs [53]. Here, to predict structurally conserved regions in lncTCF7 with high confidence, we used two orthogonal approaches. First, we used R-scape to predict statistically significant covarying base pairs, and then we validated the results from R-scape using TurboFold. Using this combined approach, we found a structurally conserved stem-loop (H12) in lncTCF7 (Figure 5). Interestingly, H12 is also among the regions with low Shannon entropy, a strong indication that this helix is potentially important for function (Figure 4C). The functional role of this region is yet to be determined. It is possible that this region plays a role in facilitating the binding of lncTCF7 to the SWI/SNF complex or other proteins, or it may play a role in other aspects of lncTCF7 function such as localization. We believe that our structural model and conservation information will be beneficial in guiding mutational analyses and functional assays to investigate the role of H12 in lncTCF7 function.

In conclusion, we report the secondary structure model for lncTCF7. This SHAPE-directed secondary structure allowed us to identify well-defined structural domains and conserved regions in lncTCF7. We believe that our structural model will support future studies aimed to understand the molecular mechanism and, possibly, the tertiary structure of lncTCF7. Moreover, we note that the combination of R-Scape and TurboFold will be useful in finding structurally conserved elements in other lncRNAs.

## 4. Materials and Methods

### 4.1. Plasmids and DNA Templates

A plasmid containing lncTCF7 (NR_131252.1) was custom synthesized using GeneArt (Thermo Fisher Scientific, Waltham, MA, USA). The lncTCF7 sequence was amplified using PCR and cloned into the pBlueScript (pBS) vector downstream of the T7 promoter and upstream of the BamHI restriction site. For 3S shotgun analysis, templates were generated via PCR using the full-length template. All primers used in this study are listed in Supplementary Table S1.

### 4.2. RNA Synthesis and Purification

RNA was synthesized and purified as described in Chillón et al. [34]. Briefly, plasmids were linearized using BamHI (NEB). RNA was transcribed from the linearized vector using T7 polymerase in a 100 μL reaction at 37 °C. Following 1.5 h of transcription, the reaction was treated with 4 U of Turbo DNase (Invitrogen, Waltham, MA, USA #AM2238) for 30 min, followed by treatment with 3 μL of 30 mg/mL Proteinase K (Thermo Fisher Scientific, Waltham, MA, USA #AM2542) for 30 min, both at 37 °C. The reaction was then loaded into an Amicon Ultra 0.5 mL filter with a 100 kDa cutoff (Millipore, Burlington, MA, USA #UFC510096). The RNA was buffer exchanged into a folding buffer (25 mM HEPES pH 7.4, 150 mM KCl, 1 mM EDTA). After filtration, size exclusion chromatography was performed in the folding buffer at room temperature using an Äkta Pure FPLC (General Electric, Boston, MA, USA). Full-length RNA was purified using Sephacryl S400, and fragments were purified using Superdex 200. Folding titrations were performed by varying the magnesium concentration (0, 3, 10, and 25 mM MgCl_2_) in the folding buffer.

### 4.3. Chemical Probing

#### 4.3.1. SHAPE-MaP

SHAPE-MaP was performed as described before [39]. Briefly, RNA was freshly purified using size exclusion chromatography in buffer containing 25 mM HEPES pH 7.4, 150 mM KCl, 1 mM EDTA with 12 mM or 25 mM MgCl_2_. After purification, RNA collected from eluted fractions was folded by incubating at 37 °C for 30 min. Probing reactions were initiated by adding 1M7 (AstaTech, Bristol, PA, USA # F51360) in DMSO at a final concentration of 10 mM or an equal volume of DMSO for control reactions and incubated at 37 °C for 10 min. After probing, RNA was purified from the probing reaction using an RNA Clean and Concentrate Kit (Zymo, Irvine, CA, USA #R1015). Modified RNA was subjected to mutational profiling as described before using lncTCF7 specific primers (Supplementary Table S1). Reverse transcription was performed using SuperScript II in a buffer containing 50 mM Tris (pH 8.0), 75 mM KCl, 6 mM MnCl_2_, 10 mM DTT and 0.5 mM dNTPs at 42 °C for 3 h. After reverse transcription, reactions were incubated at 70 °C for 15 min to inactivate SuperScript II. The cDNA was purified using G-25 columns (GE) and amplified using lncTCF7-specific primers (Supplementary Table S1). The amplicons were gel purified before library preparation using the Nextera XT kit (Illumina, San Diego, USA). High-throughput sequencing was performed at the Yale Center for Genome Analysis. Data analysis was performed using ShapeMapper (v2.1.4) with default parameters [54]. All experiments were performed in triplicate.

#### 4.3.2. DMS-MaP

For DMS probing, RNA was purified and folded in the buffer containing 125 mM cacodylic acid pH 7.0, 1M KCl, 0.5 mM EDTA, and probed with DMS in 100% ethanol at a final concentration of 10 mM or an equal volume of 100% ethanol for controls. Reverse transcription was performed using TGIRT-III enzyme in First Strand cDNA synthesis buffer [43]. The cDNA was purified using G-25 columns (General Electric, Boston, MA, USA) and amplified using lncTCF7 specific primers (Supplementary Table S1). The amplicons were gel purified before library preparation using the Nextera XT kit (Illumina, San Diego, USA). Data analysis was performed using ShapeMapper (v2.1.4) with default parameters [54]. All experiments were performed in triplicate.

#### 4.3.3. 3S Shotgun Secondary Structure Analysis

For shotgun secondary structure (3S) analysis, we used SHAPE probing followed by capillary electrophoresis to reduce high-throughput sequencing cost. RNA purification and probing were performed as described in the previous sections. SHAPE probing and capillary electrophoresis were performed as described before using FAM-labeled lncTCF7 specific primers (Supplementary Table S1). Briefly, 2 pmol of chemically modified RNA was mixed in a 12 μL annealing reaction containing 1 μL of 2 mM EDTA and 2 μL of 2 μM primer labeled with 5-FAM (see primer table). This annealing reaction was then heated to 95 °C for 2 min, placed on ice for 5 min, then incubated at 48 °C for 2 min. Once equilibrated at 48 °C, 8 μL of RT mix was added: 100 U of SuperScript III (Thermo Fisher #18080093), 4 μL of 5X First-Strand Buffer, 1 μL 100 mM DTT, 1 μL of 10 mM dNTP mix, and 1.5 μL of water. RT was carried out at 48 °C for 45 min, after which the resulting cDNA was precipitated and resuspended in formamide. cDNA fragments were sent for capillary sequencing to the DNA Analysis Facility at Science Hill at Yale University. Chromatograms were analyzed using QuShape [55]. Corresponding (+) 1M7 and (−) background (treated with DMSO) chromatograms were aligned and a normalized SHAPE reactivity for every base which was calculated. Nucleotides with high background were reported as “no data.”

### 4.4. Structure Determination and Confidence Estimation

The SHAPE-MaP directed secondary structure model of lncTCF7 was predicted using the software package RNAstructure (v 6.01.) [56]. We estimated the confidence of our SHAPE-directed model using a jackknife resampling approach [44]. First, we generated 100 “mock” datasets by randomly removing 10% of the SHAPE-MaP reactivities and labeling them as “no data.” All these “mock” data sets were then used as input to predict the secondary structure of lncTCF7 with RNAstructure. The confidence levels for each nucleotide were calculated using MATLAB.

### 4.5. Shannon Entropy Calculation

Shannon entropies for each nucleotide were calculated as previously described [45].

### 4.6. Sequence and Structure Conservation Analysis

Sequences and multiple sequence alignment of lncTCF7 from mammalian species were downloaded from the UCSC genome database using the table browser with multiz align option and the Galaxy web server [48,57,58]. We refined the multiple sequence alignment using our structural model, and the software package Infernal (v1.1.2) as previously described [53,59]. Briefly, we used the command ‘cmbuild’ to build a covariation model, followed by the command ‘cmcaliberate’ to calibrate the model. We then aligned the sequences using the calibrated covariance model with the command ‘cmalign’. Covariation analysis was performed using R-scape (v0.2.1) with the command line option “--RAFSp” [49]. TurboFold analysis was performed using the webserver [50]. The average sequence identity was calculated using R-scape.

## Supplementary Materials

Supplementary materials can be found at https://www.mdpi.com/1422-0067/20/19/4770/s1.

## Abbreviations

lncRNA	Long non-coding RNA
SHAPE	Selective 2′-Hydroxyl Acylation analyzed by Primer Extension
DMS	Dimethyl Sulfate
SEC	Size Exclusion Chromatography
WSPAR	WNT Signaling Pathway Activating Non-Coding RNA
SRA	Steroid Receptor RNA Activator
HOTAIR	Hox Transcript Antisense Interfering RNA
TCF7	Transcription Factor 7
SWI/SNF	Switch/Sucrose Non-Fermentable
3S	Shotgun Secondary Structure
MaP	Mutational Profiling
R-scape	RNA Structural Conservation Above Phylogenetic Expectation
CNBP	Cellular Nucleic Acid-Binding Protein
1M7	1-methyl-7-nitroisatoic anhydride
DMSO	Dimethyl Sulfoxide
