# Supplementary material for: Identifying Structural Domains and Conserved Regions in the Long Non-Coding RNA lncTCF7
Source: Int J Mol Sci. 2019 Sep 26;20(19):4770. doi: 10.3390/ijms20194770 (PMC6801803; doi:10.3390/ijms20194770)
Supplement: Supplementary file 1 [file ijms-20-04770-s001.pdf]

Supplementary data

Table S1. Primers

Primer	Sequence
**SHAPE-MaP Primers**	
Fwd 1	5′– CCATCCTGGCTGAACTTTGG – 3′
Rev 636	5′– CAGACGCTCACTGTCCCAC – 3′
**SHAPE probing RT primer**	
SHAPE Primer 230	5′ – AGTTAGCTCAGGTCCAAGGACTC – 3′
SHAPE Primer 452	5′ – ACTGGTGTCCATCAGGGACCAA – 3
SHAPE Primer 666	5′ – CTGGGCAGCCTCATTGTGC – 3′
**Primers used for amplifying lncTCF7 fragments**	
Fragment 1 Forward	5′ - GGCCATCCTGGCTGAACTTTGG – 3′
Fragment 1 Reverse	5′ – GTTTGGATGTGGCTGCAGGAC – 3′
Fragment 2 Forward	5′ – AAAAAATAATACGACTCACTATAGGGCATCTTCACCCCACAG – 3′
Fragment 2 Reverse	5′ – CACATGTAGGGGGTACC – 3′
Fragment 3 Forward	5′ – AAAAAATAATACGACTCACTATAGGGCTTCCAGGTTTTAGTATAGC – 3′
Fragment 3 Reverse	5′ – CTGCTGCCGTTAGG – 3′
Fragment 4 Forward	5' – AAAAAATAATACGACTCACTATAGGGACTTCTTCCAGGTTTTAGTATAG –3'
Fragment 4 Reverse	5′ – CACATGTAGGGGGTACC – 3′
Fragment 5 Forward	5′ – AAAAAATAATACGACTCACTATAGGGCTCCTCCTGCTCTAGACTGTG –3′
Fragment 5 Reverse	5′ – CACATGTAGGGGGTACC – 3′

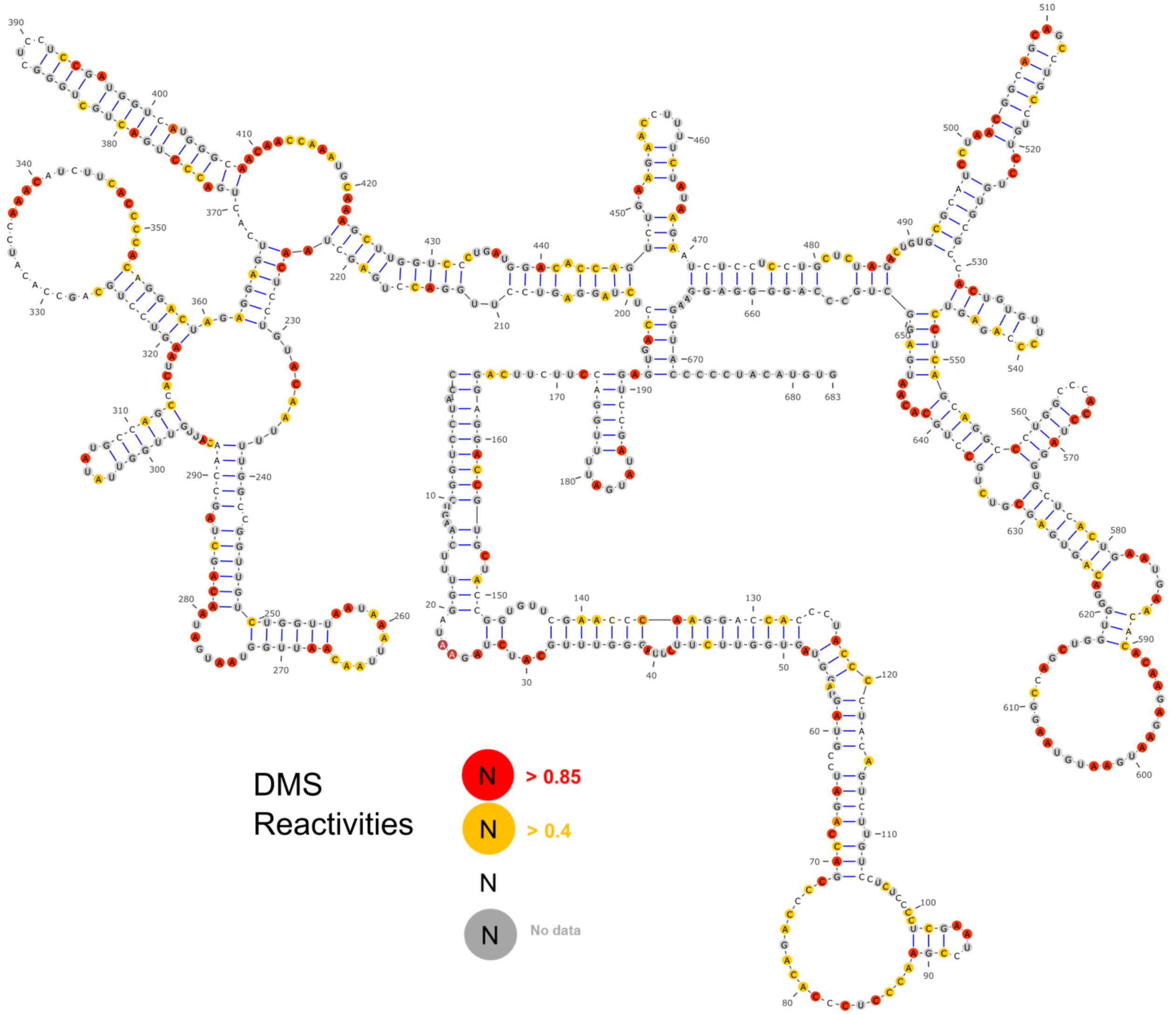

Figure S1. Secondary structure of lncTCF7 colored by DMS reactivities. lncTCF7 was probed with dimethyl sulfate (DMS) as described in Methods. Nucleotides with high SHAPE reactivity are highlighted in red, nucleotides with medium reactivity are highlighted in yellow, and nucleotides with 'no data' are highlighted in grey.
